# Antioxidant Effects of Quercetin and Naringenin Are Associated with Impaired Neutrophil Microbicidal Activity

**DOI:** 10.1155/2013/795916

**Published:** 2013-07-21

**Authors:** Francielli de Cássia Yukari Nishimura, Ana Carolina de Almeida, Bianca Altrão Ratti, Tânia Ueda-Nakamura, Celso Vataru Nakamura, Valdecir Farias Ximenes, Sueli de Oliveira Silva

**Affiliations:** ^1^Departamento de Ciências Básicas da Saúde, Universidade Estadual de Maringá, Avenida Colombo 5.790, 87020-900 Maringá, PR, Brazil; ^2^Departamento de Química, Faculdade de Ciências, Universidade Estadual Paulista, Avenida Eng. Luiz Edmundo Carrijo Coube 14-01, 17033-360 Bauru, SP, Brazil

## Abstract

Naringenin and quercetin are considered antioxidant compounds with promising activity against oxidative damage in human cells. However, no reports have described their effects on reactive oxygen species (ROS) production by phagocytes during microbicidal activity. Thus, the present study evaluated the effects of naringenin and quercetin on ROS production, specifically hypochlorous acid (HOCl), and their involvement in the microbicidal activity of neutrophils. Naringenin and quercetin inhibited HOCl production through different systems, but this inhibition was more pronounced for quercetin, even in the cell-free systems. With regard to the microbicidal activity of neutrophils, both naringenin and quercetin completely inhibited the killing of *Staphylococcus aureus*. Altogether, these data indicate that the decrease in the oxidant activity of neutrophils induced by these compounds directly impaired the microbicidal activity of neutrophils. Naringenin and quercetin exerted their effects by controlling the effector mechanisms of ROS production, with both positive and negative effects of these antioxidant agents in oxidative stress conditions and on ROS in the microbicidal activity of phagocytes. The present results challenge the traditional view of antioxidants as improvers of pathological conditions.

## 1. Introduction

Accumulating evidence indicates the involvement of reactive oxygen species (ROS) in different physiological functions and various cell signaling processes, including reproduction, cell migration, stem cell proliferation, neurogenesis, and phagocytosis [1–5]. Depending on the intracellular concentration of ROS, they can contribute to both physiological and pathological conditions. The long-term exposure of cells to enhanced levels of ROS is involved in the pathogenesis of many human diseases, including chronic inflammation, neurodegenerative disorders, and some cancers, by damaging essential molecules, such as lipids, proteins, and DNA [6–9]. Thus, maintaining an appropriate balance between ROS and antioxidant enzymes is important to avoid deleterious processes. New effective therapies based on exogenous antioxidants have been sought [10, 11]. Although the literature presents various compounds obtained from plants with promising antioxidant effects, few studies have examined the side effects of these substances on physiological functions that depend on ROS.

For example, an increasing number of studies have investigated different types of flavonoids with antioxidant potential. Most of these studies have indicated that flavonoids are promising immunomodulators, with direct antioxidant effects that involve ROS scavenging [11, 12] and anti-inflammatory effects, reflected by a reduction of the activity of ROS-forming enzymes, such as NADPH oxidase and myeloperoxidase (MPO) [13]. However, there is growing recognition of the importance of ROS, especially hypochlorous acid (HOCl), produced by phagocytes through NADPH oxidase in microbicidal activity [14, 15]. In addition to microbicidal activity, ROS produced by NADPH oxidase has emerged as an important messenger of several cellular signaling pathways, including the activation of nuclear transcription factors such as NF-*κ*B and AP-1 that are associated with physiological functions involving, respectively, inflammatory responses and the expression of protective genes that repair damaged DNA, [16].

Considering that the production of ROS by the NADPH oxidase system is an initial and critical event for the onset of oxidative stress conditions and microbicidal activity, the present study investigated the effects of quercetin and naringenin on the production of ROS, especially HOCl, and their involvement in the microbicidal activity of neutrophils. We sought to determine whether the antioxidant effects of quercetin and naringenin are associated with impaired neutrophil function.

## 2. Materials and Methods

### 2.1. Chemicals

Quercetin, naringenin, dextran, taurine, 3,3′,5,5′-tetramethylbenzidine (TMB), hydrogen peroxide, MPO, catalase, phorbol 12-myristate 13-acetate (PMA), dimethyl sulfoxide (DMSO), and Histopaque were obtained from Sigma (St. Louis, MO, USA). Naringenin and quercetin stock solutions (2.5 mM) were prepared in DMSO, stored at 8°C, and used within 1 week. Dimethyl sulfoxide was added at the same concentration in all of the samples including the controls at a final concentration of 0.2%, a concentration that has been shown to not affect neutrophil viability.

### 2.2. Neutrophils and Total Leukocytes

Neutrophils and total leukocytes were isolated from peripheral venous blood obtained from healthy volunteers by centrifugation over a Ficoll-Hypaque gradient (Histopaque; *d* = 1.077) [17, 18]. Cell concentration and viability were determined in a Neubauer chamber. Neutrophils (2.5 × 10^6^ cells/mL) and total leukocytes (2.0 × 10^6^ cells/mL) were suspended in 10 mM phosphate-buffered saline (PBS; pH 7.4) supplemented with 1 mg/mL glucose, 1 mM CaCl_2_, and 0.5 mM MgCl_2_.

### 2.3. Effects of Quercetin and Naringenin on HOCl Production

The concentration of HOCl produced in cellular and cell-free systems was evaluated according to the method described by Dypbukt et al. [19]. Briefly, HOCl was trapped as the less reactive and stable taurine chloramine. Taurine chloramine in the supernatant was then quantified by the oxidation of TMB (10 mM in 1 : 1 [v/v] dimethylformamide/0.8 M acetic acid, containing 100 *μ*M potassium iodide) to a blue product with maximum absorbance at 655 nm. A calibration curve that consisted of pure HOCl was generated to calculate the production of the oxidant. The analyses were performed in a final volume of 250 *μ*L using a microplate reader spectrophotometer (Biotec power-WaveX5, USA). The effects of naringenin and quercetin on HOCl production were studied using three different experimental models.

#### 2.3.1. Cell-Free System: Antioxidant Effects of Naringenin and Quercetin

A 96-well culture plate that contained 50 *μ*M HOCl and 5 mM taurine in supplemented PBS was incubated in the presence or absence of naringenin and quercetin (25 and 50 *μ*M) for 10 min at 25°C. The final volume was 200 *μ*L, and the reactions were triggered by adding HOCl. The TMB solution (50 *μ*L) was then added to measure the remaining taurine chloramine.

#### 2.3.2. Cell-Free System: Effects of Naringenin and Quercetin on HOCl Production by MPO/Hydrogen Peroxide (H_2_O_2_)/Cl^−^

A 96-well culture plate that contained MPO (65 nM), 5 mM taurine, and 50 *μ*M H_2_O_2_ in supplemented PBS was incubated in the presence or absence of quercetin and naringenin (25 and 50*μ*M) for 10 min at 25°C. The reactions were triggered by adding H_2_O_2_ and stopped by adding catalase (65 *μ*g/mL). The final volume was 200 *μ*L. The TMB solution (50 *μ*L) was then added to the samples, and HOCl production was quantified. The positive control, without the tested substances, was used to calculate the inhibitory effect.

#### 2.3.3. Effects of Naringenin and Quercetin on HOCl Production by Neutrophils

Neutrophils (1.0 × 10^6^ cells/mL) were preincubated in the presence or absence of quercetin and naringenin (25 and 50 *μ*M) in supplemented PBS that contained 5 mM taurine for 10 min at 37°C. All of the samples were then incubated with PMA (6.65 *μ*g/mL) for 30 min at 37°C. Afterward, the reactions were stopped by adding catalase (65 *μ*g/mL). The final volume was 200 *μ*L. The cells were pelleted by centrifugation (1,200 ×g for 10 min at 24°C), and HOCl production was quantified as stated previously.

### 2.4. Effects of Naringenin and Quercetin on ROS Production by Leukocytes

Dihydrorhodamine 123 (DHR) is widely used for the detection of intracellular oxidant species production by cell systems [20]. The oxidation of DHR by ROS results in the formation of rhodamine, a highly fluorescent component. Total leukocytes (2.0 × 10^6^ cells/mL) were incubated with quercetin or naringenin (10 and 100 *μ*M) for 2 h and then stimulated with PMA (400 nM) for 10 min. After PMA stimulation, the cells were incubated with DHR (10 mg/mL) for 5 min, washed once with PBS, and suspended in PBS/bovine serum albumin/azide buffer. The fluorescence of gated neutrophils was detected at FL1, counting 30,000 events/gate, in a FACS Canto Flow Cytometer (BD, Franklin Lakes, NJ, USA). The data were analyzed using Flow Cytometry Analysis software (Treestar, Ashland, OR, USA), and the results are expressed as the fluorescence intensity and percentage of positive cells in the sample.

### 2.5. Effects of Naringenin and Quercetin on Microbicidal Assay

#### 2.5.1. Growth and Opsonization of Bacteria

*Staphylococcus aureus* (ATCC-25923) was grown overnight on nutrient agar plates at 37°C. The cell colonies were scraped and suspended in sterile PBS (10 mM), and the number of viable cells was estimated by measuring the optical density at 550 nm (OD_550_) using suitable calibration curves (MacFarland scale). Bacteria (2.0 × 10^7^ cells/mL) were opsonized with 10% serum (v/v, final concentration) from healthy donors for 30 min at 37°C with constant and moderate agitation and used for the killing assay.

#### 2.5.2. Bacterial Killing

Neutrophils (2.0 × 10^6^ cells/mL per assay) were suspended in RPMI 1640 and incubated with opsonized bacteria (2.0 × 10^7^ cells/mL) in a final volume of 1.0 mL. Killing activity was monitored in the presence or absence of quercetin and naringenin (25 and 50 *μ*M). The samples were maintained at 37°C with moderate shaking. Killing activity was determined by aseptically removing the samples at intervals of 0, 30, 60, 90, and 120 min. These samples were then diluted in sterile distilled water (1 : 10), whirlmixed for 5 min to lyse neutrophils, and subsequently diluted in sterile saline (1 : 500). The number of viable bacteria was evaluated by spread-plating suitable diluted samples on nutrient agar and incubating them at 37°C for 24 h [21].

### 2.6. Statistical Analysis

Comparisons were made using one-way analysis of variance (ANOVA) and the Dunnett multiple comparisons test. The results are expressed as the mean ± standard error of the mean (SEM) of at least three independent experiments. The data were analyzed using BioEstat 5.0 software. Values of *P* < 0.05 were considered statistically significant. 

## 3. Results 

The present study investigated the antioxidant activity of naringenin and quercetin in three different systems of HOCl formation.

### 3.1. Cell-Free System: Direct HOCl Antioxidant Effects of Naringenin and Quercetin

**Figure 1:**
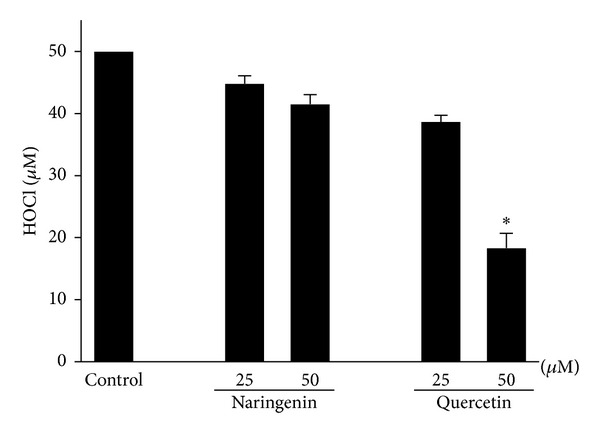
Antioxidant effects of naringenin and quercetin on HOCl. A 96-well culture plate that contained HOCl (50 *μ*M) and taurine (5 mM) was incubated in the presence or absence of naringenin and quercetin (25 and 50 *μ*M). The TMB solution (50 *μ*L) was then added to the samples, and HOCl was quantified. The data are expressed as the mean ± SEM of three experiments. *P < 0.05, compared with control group.

In our first cell-free system, quercetin but not naringenin functioned as a HOCl scavenger. The scavenging action of quercetin depended on its concentration. Quercetin at a concentration of 50 *μ*M decreased HOCl by greater than 50% compared with the control group (Figure 1).

### 3.2. Cell-Free System: Effects of Naringenin and Quercetin on HOCl Production by MPO/H_2_O_2_/Cl^−^

**Figure 2:**
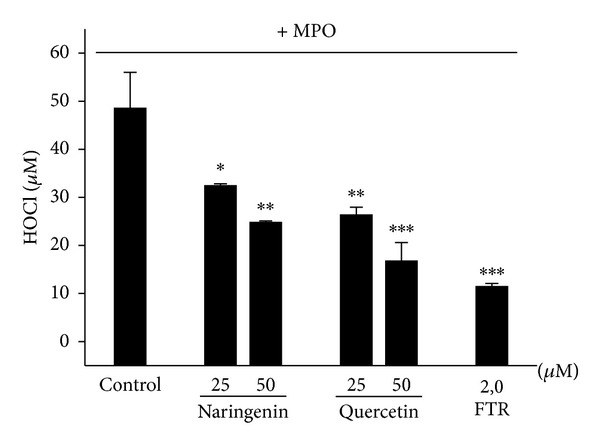
Effects of naringenin and quercetin on myeloperoxidase (MPO)-HOCl production. A 96-well culture plate that contained MPO (65 nM), taurine (5 mM), and H_2_O_2_ (50 *μ*M) was incubated in the presence or absence of naringenin and quercetin (25 and 50 *μ*M). The TMB solution (50 *μ*L) was then added to the samples, and HOCl production was quantified. The positive control (i.e., without the tested substances) was used to calculate the inhibitory effect. The data are expressed as the mean ± SEM of three experiments. *P < 0.05, **P < 0.005, and ***P < 0.001, compared with control group.

We also evaluated the effects of naringenin and quercetin in a cell-free system that contained MPO/H_2_O_2_/Cl^−^. In this experimental model, HOCl is directly produced by the enzymatic system. Both quercetin and naringenin significantly and dose-dependently decreased HOCl production compared with the control group (Figure 2). Naringenin was less effective than quercetin in inhibiting HOCl formation, but this difference was not significant. A decrease in HOCl production by more than 60% was observed with the higher concentration of quercetin (50 *μ*M), whereas the decrease induced by naringenin was approximately 50% at the same concentration. One sample with 5-fluortryptamine (FTR), an MPO inhibitor [22], was assayed to compare the potential of the flavonoids as inhibitors of the chlorinating activity of MPO. The flavonoids were less effective than FTR.

### 3.3. Effects of Naringenin and Quercetin on HOCl Production by Neutrophils

**Figure 3:**
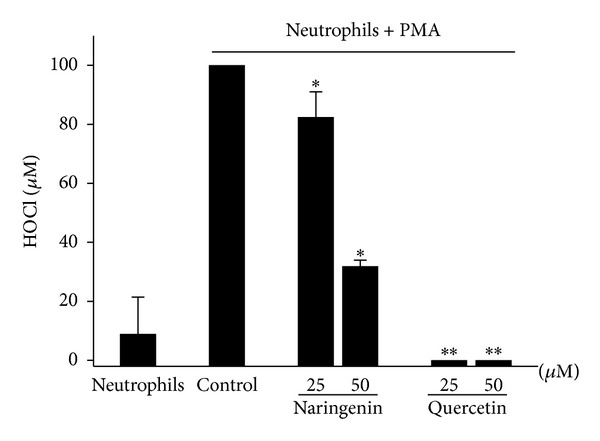
Effects of naringenin and quercetin on HOCl production by activated neutrophils. Neutrophils (1.0 × 10^6^ cells/mL) were preincubated in the presence or absence of naringenin and quercetin (25 and 50 *μ*M) with 5 mM taurine. All of the samples were then incubated with PMA (6.65 *μ*g/mL). The TMB solution (50 *μ*L) was then added to the samples, and HOCl production was quantified. The data are expressed as the mean ± SEM of three experiments. *P < 0.05,**P < 0.01 compared with control group.

To better understand the effects of naringenin and quercetin on HOCl formation, we assessed the effects of these compounds in a third system, PMA-activated neutrophils. As expected, both compounds inhibited HOCl production compared with the control (Figure 3). Quercetin exerted a strong inhibitory effect at both concentrations tested, causing approximately 100% decreases in HOCl production. However, naringenin exerted a significant effect only at 50 *μ*M, inhibiting HOCl production by approximately 60%.

### 3.4. Effects of Naringenin and Quercetin on ROS Production by Leukocytes

**Figure 4:**
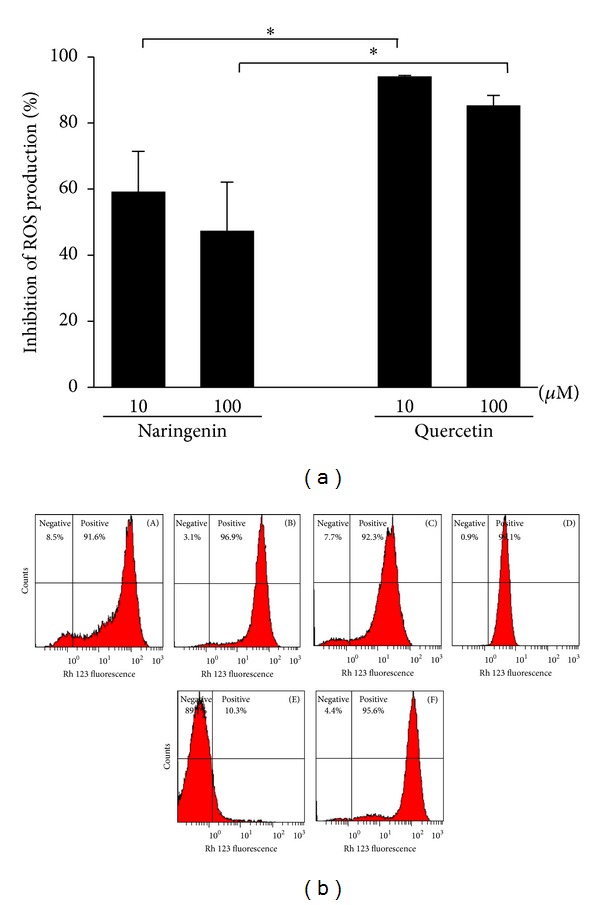
Effects of naringenin and quercetin on ROS production by neutrophils. (a) Neutrophils were preincubated in the presence or absence of naringenin and quercetin (10 and 100 *μ*M) and stimulated with PMA (400 nM), and intracellular ROS production was determined by flow cytometry using DHR as a probe. (b) Representative histograms are shown in logarithmic scale. Neutrophils were preincubated in the presence of naringenin 10 *μ*M (A) and 100 *μ*M (B) and activated with PMA. Neutrophils were preincubated in the presence of quercetin 10 *μ*M (C) and 100 *μ*M (D) and activated with PMA. Neutrophils (negative control) (E); neutrophils activated with PMA (positive control) (F). The percentage of inhibition of ROS production by naringenin and quercetin was calculated and compared with the positive group. The data are expressed as the mean ± SEM of four experiments. *P < 0.05, 10 *μ*M naringenin versus 10 *μ*M quercetin and 100 *μ*M naringenin versus 100 *μ*M quercetin.

As a second step, we compared the ability of quercetin and naringenin to inhibit intracellular ROS production assessed by flow cytometry, in which the nonfluorescent DHR is oxidized by ROS, producing fluorescent rhodamine. Again, quercetin was more efficient than naringenin (Figure 4). Quercetin at both tested concentrations inhibited ROS by more than 80%. Naringenin at higher concentrations inhibited ROS by approximately 50%.

### 3.5. Effects of Naringenin and Quercetin on Microbicidal Activity

**Figure 5:**
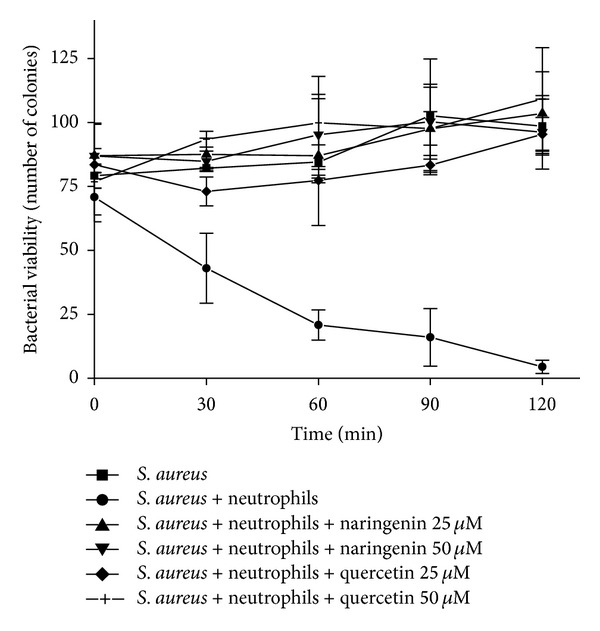
Effects of naringenin and quercetin on the kinetics of the killing of *S. aureus* by neutrophils. Neutrophils (2.0 × 10^6^ cells/mL per assay) were incubated with opsonized bacteria (2.0 × 10^7^ cells/mL). Microbicidal activity was monitored in the presence and absence of 25 *μ*M (▲) and 50 *μ*M (*▼*) naringenin and 25 *μ*M (♦) and 50 *μ*M (+) quercetin. Killing activity was determined by aseptically removing the samples at intervals of 0, 30, 60, 90, and 120 min. The number of viable bacteria was evaluated by spread-plating suitable diluted samples on nutrient agar. The controls included bacteria alone (■) and bacteria plus neutrophils (●). The data are expressed as the mean ± SEM of three experiments.

We showed that quercetin and, to a lesser extent, naringenin affected HOCl production by PMA-activated neutrophils. HOCl is a toxic metabolite responsible for the microbicidal activity of phagocytes [14, 15]. We expected that these compounds would have different effects on neutrophil microbicidal activity. Thus, we studied the effects of these flavonoids on the microbicidal activity of neutrophils by spread-plating *S. aureus* onto a nutrient-agar medium after incubation with neutrophils. In contrast to the previous results of the present study mentioned above, both quercetin and naringenin (25 and 50 *μ*M) completely inhibited neutrophil microbicidal activity compared with the control group (Figure 5).

## 4. Discussion

Numerous compounds with potential antioxidant effects and promising activity against many human diseases associated with oxidative damage have been studied over the past years [23, 24]. These compounds include naringenin and quercetin, two flavonoids with antioxidant effects that act as ROS scavengers [11, 12] and inhibit the activity of ROS-forming enzymes (e.g., NADPH oxidase) [13]. However, no reports have described their action on the microbicidal response of neutrophils. The microbicidal activity of phagocytes is well known to depend on ROS, and HOCl plays an important role in this process [25]. The present study sought to further elucidate the effects of naringenin and quercetin on the neutrophil response, especially with regard to microbicidal activity.

We first investigated the antioxidant effects of naringenin and quercetin on HOCl production by cellular and cell-free systems. The effects of naringenin observed in the MPO model and in PMA-activated neutrophils, compared with the first cell-free system, indicated that significant inhibition of MPO chlorinating activity can be induced by this compound. The production of HOCl might be reduced in the presence of flavonoids that act as MPO inhibitors [26]. Thus, the 15% (cell-free system) to 50% (MPO system) increases in the inhibition of HOCl production induced by 50 *μ*M naringenin might be related to direct inhibition of MPO chlorinating activity. Quercetin exhibited the same pattern of inhibition in the cell-free systems, suggesting that quercetin is a better scavenger of HOCl and poor inhibitor of MPO. Quercetin also markedly inhibited HOCl production in PMA-activated neutrophils. In this system, PMA activated the NADPH oxidase complex, which is responsible for the production of superoxide anions and, after a cascade of reactions, produces H_2_O_2_ and HOCl [27]. Therefore, quercetin, a well-known antioxidant, could react with all ROS formed in the cellular system, consequently disrupting HOCl formation through a scavenging effect [11, 12]. To support this possibility, the DHR assay confirmed that quercetin is an efficient scavenger of ROS generated by activated neutrophils.

We showed that naringenin and quercetin also inhibited the microbicidal activity of neutrophils. These results may reflect the antioxidant activity of these compounds, which consume both HOCl and ROS precursors of HOCl, thus inhibiting the formation of HOCl derivatives with high microbicidal activity, such as singlet oxygen. The suppression of these ROS induced a direct effect on microbicidal activity [28].

In conclusion, the flavonoids naringenin and quercetin exert their effects by controlling the effector mechanisms of ROS production, which might be seen as a positive effect when considering the importance of antioxidant agents in oxidative stress conditions or a negative effect when considering the importance of ROS for microbicidal activity. The latter interpretation is the highlight of the present study. Previous studies suggested that an increase in basal antioxidant capacity can contribute to the development of certain cancers [9, 29]. DeNicola et al. [30] provided evidence that several oncogenes actively upregulate physiological antioxidant enzymes, promoting a ROS detoxification program that is required for tumor initiation. These results challenge the traditional view that the greater intake of antioxidants is always associated with improvements in pathological conditions [31, 32]. This view must be revisited, especially with regard to infectious diseases and the development of certain cancers.

## References

[1] Morgan MJ, Liu Z-G (2010). Reactive oxygen species in TNF*α*-induced signaling and cell death. *Molecules and Cells*.

[2] Dickinson BC, Chang CJ (2011). Chemistry and biology of reactive oxygen species in signaling or stress responses. *Nature Chemical Biology*.

[3] Rizzo A, Roscino MT, Binetti F, Sciorsci RL (2012). Roles of reactive oxygen species in female reproduction. *Reproduction in Domestic Animals*.

[4] Kim J-S, Huang TY, Bokoch GM (2009). Reactive oxygen species regulate a slingshot-cofilin activation pathway. *Molecular Biology of the Cell*.

[5] Dickinson BC, Peltier J, Stone D, Schaffer DV, Chang CJ (2011). Nox2 redox signaling maintains essential cell populations in the brain. *Nature Chemical Biology*.

[6] Chapman ALP, Hampton MB, Senthilmohan R, Winterbourn CC, Kettle AJ (2002). Chlorination of bacterial and neutrophil proteins during phagocytosis and killing of *Staphylococcus aureus*. *The Journal of Biological Chemistry*.

[7] Frey RS, Ushio-Fukai M, Malik AB (2009). NADPH oxidase-dependent signaling in endothelial cells: role in physiology and pathophysiology. *Antioxidants & Redox Signaling*.

[8] Correia SC, Santos RX, Perry G, Zhu X, Moreira PI, Smith MA (2012). Mitochondrial importance in Alzheimer’s, Huntington’s and Parkinson’s diseases. *Advances in Experimental Medicine and Biology*.

[9] Lonkar P, Dedon PC (2011). Reactive species and DNA damage in chronic inflammation: reconciling chemical mechanisms and biological fates. *International Journal of Cancer*.

[10] Tiwari AK (2004). Antioxidants: new-generation therapeutic base for treatment of polygenic disorders. *Current Science*.

[11] Ursini F, Maiorino M, Morazzoni P, Roveri A, Pifferi G (1994). A novel antioxidant flavonoid (IdB 1031) affecting molecular mechanisms of cellular activation. *Free Radical Biology and Medicine*.

[12] Agati G, Azzarello E, Pollastri S, Tattini M (2012). Flavonoids as antioxidants in plants: location and functional significance. *Plant Science*.

[13] Ciz M, Denev P, Kratchanova M, Vasicek O, Ambrozova G, Lojek A (2012). Flavonoids inhibit the respiratory burst of neutrophils in mammals. *Oxidative Medicine and Cellular Longevity*.

[14] Wang L, Bassiri M, Najafi R (2007). Hypochlorous acid as a potential wound care agent. *Journal of Burns and Wounds*.

[15] Gebicka L, Banasiak E (2012). Hypochlorous acid-induced heme damage of hemoglobin and its inhibition by flavonoids. *Toxicology in Vitro*.

[16] Valko M, Leibfritz D, Moncol J, Cronin MTD, Mazur M, Telser J (2007). Free radicals and antioxidants in normal physiological functions and human disease. *The International Journal of Biochemistry and Cell Biology*.

[17] Bøyum A (1976). Isolation of lymphocytes, granulocytes and macrophages. *Scandinavian Journal of Immunology*.

[18] Böyum A (1968). Isolation of leucocytes from human blood. A two-phase system for removal of red cells with methylcellulose as erythrocyte-aggregating agent. *Scandinavian Journal of Clinical and Laboratory Investigation, Supplement*.

[19] Dypbukt JM, Bishop C, Brooks WM, Thong B, Eriksson H, Kettle AJ (2005). A sensitive and selective assay for chloramine production by myeloperoxidase. *Free Radical Biology and Medicine*.

[20] Crow JP (1997). Dichlorodihydrofluorescein and dihydrorhodamine 123 are sensitive indicators of peroxynitrite in vitro: implications for intracellular measurement of reactive nitrogen and oxygen species. *Nitric Oxide: Biology and Chemistry*.

[21] Hampton MB, Winterbourn CC (1999). Methods for quantifying phagocytosis and bacterial killing by human neutrophils. *Journal of Immunological Methods*.

[22] Zeraik ML, Ximenes VF, Regasini LO (2012). 4′-Aminochalcones as novel inhibitors of the chlorinating activity of myeloperoxidase. *Current Medicinal Chemistry*.

[23] Yagi H, Tan J, Tuan RS (2012). Polyphenols suppress hydrogen peroxide-induced oxidative stress in human bone-marrow derived mesenchymal stem cells. *Journal Cellular Chemistry*.

[24] Zhang M, Swarts SG, Yin L (2011). Antioxidant properties of quercetin. *Advances in Experimental Medicine and Biology*.

[25] Winterbourn CC, Hampton MB, Livesey JH, Kettle AJ (2006). Modeling the reactions of superoxide and myeloperoxidase in the neutrophil phagosome: implications for microbial killing. *The Journal of Biological Chemistry*.

[26] Kostyuk VA, Kraemer T, Sies H, Schewe T (2003). Myeloperoxidase/nitrite-mediated lipid peroxidation of low-density lipoprotein as modulated by flavonoids. *FEBS Letters*.

[27] Robinson JM (2008). Reactive oxygen species in phagocytic leukocytes. *Histochemistry and Cell Biology*.

[28] Winterbourn CC, Kettle AJ (2013). Redox reactions and microbial killing in the neutrophil phagosome. *Antioxidants & Redox Signaling*.

[29] Trachootham D, Alexandre J, Huang P (2009). Targeting cancer cells by ROS-mediated mechanisms: a radical therapeutic approach?. *Nature Reviews Drug Discovery*.

[30] DeNicola GM, Karreth FA, Humpton TJ (2011). Oncogene-induced Nrf2 transcription promotes ROS detoxification and tumorigenesis. *Nature*.

[31] Bischoff SC (2008). Quercetin: potentials in the prevention and therapy of disease. *Current Opinion in Clinical Nutrition and Metabolic Care*.

[32] Xiao Z-P, Peng Z-Y, Peng M-J, Yan W-B, Ouyang Y-Z, Zhu H-L (2011). Flavonoids health benefits and their molecular mechanism. *Mini-Reviews in Medicinal Chemistry*.

